# Correlation among semiquantitative High‐Resolution Computed Tomography severity scores and clinical and biochemical parameters in COVID‐19, is it really effective?

**DOI:** 10.1111/crj.13567

**Published:** 2022-12-26

**Authors:** Chiara Zanon, Giulio Cabrelle, Filippo Crimì, Emilio Quaia

**Affiliations:** ^1^ Institute of Radiology, Department of Medicine DIMED Padua University‐Hospital Padua Italy

To the Editor in Chief

We read with great interest the recently published study by Komurcuoglu et al.,^1^ “Correlation between chest CT severity scores and clinical and biochemical parameters of COVID‐19 pneumonia.” This study aims to summarize the correlation between thoracic high‐resolution computerized tomography (HRCT) severity scores and clinical and biochemical parameters in patients who tested positive for COVID‐19.

The authors should be commended for their efforts in evaluating clinical and biochemical parameters with HRCT findings of COVID‐19 in such a large population.

We agree that semiquantitative radiological scoring systems might be helpful for radiologists in detecting and classifying medical images by discovering hidden patterns and abnormal conditions. In the era of personalized medicine, potentially more accurate methods for the imaging characterization of a specific disease, beyond “classical” risk factors and stage, are doubtless attractive.

Many studies have widely underlined the role of chest CT as a fundamental diagnostic tool for diagnosing COVID‐19 infection.^2^ HRCT is a diagnostic method used frequently to detect lung involvement and prevalence of the disease, especially in diagnosing PCR‐negative cases.^3^ Especially at the beginning of the pandemics, chest CT proved helpful because of the typical signs of COVID‐19 pneumonia that could be easily identified.

However, some concerns could be raised, especially about the inclusion criteria adopted in this study which, in our opinion, suggest taking with caution at least some of the conclusions drawn by the Authors.

The authors analyzed 277 patients classified as COVID‐19 positive by at least two positive RT‐PCR from nasopharyngeal swabs and HRCT, consistent with pneumonia.

First of all, the precise timing among the onset of symptoms, RT‐PCR, and HRCT was not reported, and this could have biased the score's severity. At the beginning of the disease, patients tend to show mainly ground‐glass opacities, while in more advanced stages, they have more severe lung patterns like crazy‐paving and consolidations.^2^ The accurate specificity of the HRCT scan could be even higher than that reported because the presence of mono‐lateral lung involvement at the CT scan was never specified.

Concerning the age distribution, a significant skewness toward older ages can be appreciated, given that 80% of patients were older than 64 and, therefore, a more vulnerable population. Moreover, there are no specifications about the type of coexisting comorbidities since the severity of COVID‐19 pneumonia could have been related to many pathologies, above all cardiovascular disease.^4^

In our opinion, images should have been reviewed by at least two expert radiologists for more reliable detection of radiological signs in HRCTs. This method should have been reported in the study's limitations.

COVID‐19 pneumonia was associated to post‐infective pulmonary fibrosis^5^; that's why a longer follow‐up could have added more useful information together to the patient's drug therapy (before and during infection), potentially related to outcomes.

In summary, while we agree with the conclusions that the correlation between HRCT severity scores and clinical and biochemical parameters is an essential factor for determining the severity and prognosis in COVID‐19 patients, it should be highlighted that the multiple biases of this study could affect the robustness of the conclusions drawn by the authors.

## CONFLICT OF INTEREST

The authors declare no conflict of interest.
